# Risk stratification after acute myocardial infarction by amplitude–frequency mapping of cyclic variation of heart rate

**DOI:** 10.1111/anec.12825

**Published:** 2021-02-01

**Authors:** Junichiro Hayano, Norihiro Ueda, Masaya Kisohara, Emi Yuda, Eiichi Watanabe, Robert M. Carney, James A. Blumenthal

**Affiliations:** ^1^ Nagoya City University Graduate School of Medical Sciences Nagoya Japan; ^2^ Tohoku University Graduate School of Engineering Sendai Japan; ^3^ Department of Cardiology Fujita Health University Bantane Hospital Nagoya Japan; ^4^ Department of Psychiatry Washington University School of Medicine St Louis MI USA; ^5^ Department of Psychiatry Duke University Medical Center Durham NC USA

**Keywords:** ALLSTAR, cyclic variation of heart rate, heart rate variability, mortality, myocardial infarction, sleep apnea

## Abstract

**Background:**

Blunted cyclic variation of heart rate (CVHR), measured as a decrease in CVHR amplitude (Acv), predicts mortality risk after acute myocardial infarction (AMI). However, Acv also can be reduced in mild sleep apnea with mild O_2_ desaturation. We investigated whether Acv's predictive power for post‐AMI mortality could be improved by considering the effect of sleep apnea severity.

**Methods:**

In 24‐hr ECG in 265,291 participants of the Allostatic State Mapping by Ambulatory ECG Repository project, sleep apnea severity was estimated by the frequency of CVHR (Fcv) measured by an automated algorithm for auto‐correlated wave detection by adaptive threshold (ACAT). The distribution of Acv on the Acv–Fcv relation map was modeled by percentile regression, and a function converting Acv into percentile value was developed. In the retrospective cohort of the Enhancing Recovery in Coronary Heart Disease (ENRICHD) study, consisting of 673 survivors and 44 non‐survivors after AMI, the mortality predictive power of percentile Acv calculated by the function was compared with that of unadjusted Acv.

**Results:**

Among the ALLSTAR ECG data, low Acv values appeared more likely when Fcv was low. The logistic regression analysis for mortality in the ENRICHD cohort showed c‐statistics of 0.667 (*SE*, 0.041), 0.817 (0.035), and 0.843 (0.030) for Fcv, unadjusted Acv, and the percentile Acv, respectively. Compared with unadjusted Acv, the percentile Acv showed a significant net reclassification improvement of 0.90 (95% CI, 0.51–1.42).

**Conclusions:**

The predictive power of Acv for post‐AMI mortality is improved by considering its relation to sleep apnea severity estimated by Fcv.

## 1 INTRODUCTION

Cyclic variation of heart rate (CVHR) is a characteristic pattern of heart rate accompanying sleep apnea and hypopnea episodes; it consists of bradycardia during the apnea–hypopnea period and tachycardia upon its cessation (Guilleminault et al., 1984). The frequency of CVHR (Fcv) during sleep correlates with the apnea–hypopnea index (AHI) and is used as an indicator for ECG screening of sleep‐disordered breathing (Hayano et al., 2013; Hayano, Watanabe, et al., 2011). Furthermore, blunted CVHR measured as a decrease in CVHR amplitude (Acv) is one of the most powerful ECG‐derived predictors of mortality risk in patients after acute myocardial infarction (AMI; Hayano et al., 2017). CVHR is known to reflect an increase in heart rate primarily caused by vagal withdrawal at the termination of each apnea/hypopnea episode (Guilleminault et al., 1984; Zwillich et al., 1982), and thus, a decrease in Acv is believed to reflect cardiac vagal dysfunction associated with a higher baseline heart rate during sleep (Hayano et al., 2017). However, polysomnographic observations show that the lowest values of Acv are more likely to appear in mild sleep apnea, particularly when accompanying O_2_ desaturation is mild. From these observations, we hypothesized that the predictive power of decreased Acv could be improved by considering the relation of Acv to sleep apnea severity.

To test this hypothesis, we conducted this study in three steps, Step 1: Analysis of the relationships of Acv with sleep apnea severity and O_2_ desaturation, Step 2: analysis of Acv distribution in an Acv–Fcv relationship map and the development of a model to convert Acv into a percentile value as functions of Fcv, and Step 3: evaluation of the improved predictive power of Acv for post‐AMI mortality by applying the converting functions. We used a polysomnogram database of patients with suspected sleep‐disordered breathing for Step 1, 24‐hr ECG big data of patients with suspected or diagnosed cardiac diseases for Step 2, and independent 24‐hr ECG data from a retrospective cohort of post‐AMI patients for Step 3. We sought to determine whether the use of percentile Acv adjusted for the effects of sleep apnea severity improves the mortality predictive power of Acv.

## 2 METHODS

### 2.1 Step 1: Analysis of polysomnographic data

To investigate the correlates of Acv, the associations between Acv, sleep apnea severity, and O_2_ desaturation were examined using a database of all‐night polysomnograms in 862 participants. The study use of this database has been approved by the institutional review board of the Fujita Health University, Toyoake, Aichi, Japan (No. 09‐008), and by the Ethics Review Committee of the Nagoya City University Graduate School of Medical Sciences, Nagoya, Japan (No. 390).

The characteristics of this database have been reported previously (Hayano, Watanabe, et al., 2011). Briefly, the participants were selected from 1,193 consecutive participants referred to the Sleep Laboratory of the Fujita Health University Hospital between January 2005 and December 2008 for diagnostic evaluation of suspected sleep‐disordered breathing. Participants were excluded if they (a) were < 16 yr of age, (b) had an implanted pacemaker, (c) had persistent atrial fibrillation, or (d) had a total length of analyzable ECG in the polysomnographic recording <360 min. The polysomnograms were recorded using the Alice 3 and Alice 4 Diagnostic Sleep System (Philips Respironics). Sleep stages and respiratory events were scored according to the AASM Manual for the Scoring of Sleep and Associated Events (Berry et al., 2015). The average hourly frequency of apneic and hypopneic episodes was measured as AHI. O_2_ desaturation was measured for individual apneic and hypopneic episodes and averaged over all episodes during the night. Time in bed instead of total sleep time was used as the denominator in the calculation of indices to conform with the calculation of Fcv.

### 2.2 Step 2: Analysis of ECG big data

To analyze the relationship between Acv and Fcv, we used big data of 24‐hr ECG from the Allostatic State Mapping by Ambulatory ECG Repository (ALLSTAR) project (Hayano et al., 2020; Yuda et al., 2021). The ALLSTAR database contained 430,169 Holter ECG data recorded from patients throughout Japan between November 2007 and March 2014. The ALLSTAR project was approved by the Ethics Review Committee of Nagoya City University Graduate School of Medical Sciences (No. 709). Also, following the Ethical Guidelines for Medical and Health Research Involving Human Subjects (by the Ministry of Education, Culture, Sports, Science and Technology and the Ministry of Health, Labor and Welfare, Japan, December 22, 2014), the purpose and information utilized in this project were made publicly available through the project's homepages (http://www.suzuken.co.jp/product/holter/detail/ and http://www.med.nagoya‐cu.ac.jp/mededu.dir/allstar/), in which opportunities to refuse the use of personal data were ensured for all research participants.

The 24‐hr ECG data were recorded from patients by their respective physicians for clinical indication. The clinical characteristics of patients of this database have been reported elsewhere (Yuda et al., 2021). All data were recorded with the Cardy series of Holter ECG recorders (Cardy 2, Cardy 2P, Cardy 203, Cardy 301, Cardy 302 Mini and Max, Cardy 303 pico, and Cardy 303 pico+, SUZUKEN Co., Ltd.), by which multi‐channel ECG data were digitized at 125 Hz with a 10‐bit resolution (0.02 mV/digit). The data were referred to ECG analysis centers (SUZUKEN Co., Ltd.), analyzed with Holter ECG analyzers (Cardy Analyzer 05, SUZUKEN Co., Ltd.), and reviewed by experienced medical technologist under supervisions by contracted cardiologists.

For the present study, 24‐hr ECG data were included in the study only if all the following conditions were met:

1. Participant age at ECG recording was >20 years;
2. The first ECG recording was used, if there was a repeated recording; and
3. Data were recorded for >3 hr between 23:00 and 06:00.

Data were excluded if ECG showed at least one of the following:

1. Evidence of artificial pacemaker implantation; or
2. Non‐sinus rhythm beats >20% of total recorded beats between 23:00 and 06:00.

### 2.3 Step 3: Application to post‐AMI cohort data

To examine whether the conversion to percentile values improves the mortality predictive power of Acv, we used retrospective cohort data from a subset of patients from the Enhancing Recovery in Coronary Heart Disease (ENRICHD) study (Berkman et al., 2003). The data consisted of patients who had an AMI, were at elevated psychological risk for adverse events because they were either depressed or had low social support, and were admitted to the coronary care units of four of the eight ENRICHD clinical trial sites (Washington University, St. Louis, Missouri; Duke University, Durham, North Carolina; Harvard University, Boston, Massachusetts; and Yale University, New Haven, Connecticut) between October 1997 and January 2000. The details of this cohort have reported elsewhere (Hayano et al., 2012; Hayano, Kiyono, et al., 2011). The collection and analysis of Holter ECG recordings were approved by the ethics committees of the respective clinical sites. All participants provided written informed consent to participate in the study.

Holter ECG was recorded by Marquette Model 8,500 monitors at each site. Holter recordings were scanned at the Heart Rate Variability Core laboratory at Washington University on a Marquette SXP Laser scanner with software version 5.8 (Marquette Electronics) using standard procedures. The end‐point analyzed in the present study was all‐cause mortality. The patients were followed for up to 30 months. During this period, 617 patients survived and 43 (6.5%) patients died from all causes.

Patients were included in this report if they had analyzable Holter ECG data for > 3 hr of time in bed. Patients were excluded if they: (a) had other life‐threatening illnesses; (b) were too ill or logistically unable to participate; (c) had ECG data in sinus rhythm < 80% of total recorded beats during sleep period; or (d) had atrial fibrillation, atrial flutter, or an implanted pacemaker or defibrillator.

### 2.4 Detection of CVHR

From the ECG recordings of polysomnogram data, ALLSTAR big data, and ENRICHD cohort data, CVHR was detected by an automated algorithm for auto‐correlated wave detection with adaptive threshold (ACAT; Hayano, Watanabe, et al., 2011). The details of the ACAT algorithm have been reported elsewhere (Hayano et al., 2012, 2013; Hayano, Watanabe, et al., 2011). Briefly, all R waves were detected and R–R interval time series were generated only using intervals between consecutive sinus rhythm R waves (N–N interval). The N–N interval time series were interpolated with a horizontal‐step function, resampled at 2 Hz, and smoothed by second‐order polynomial fitting. All dips in the smoothed trend with widths between 10 and 120 s and depth‐to‐width ratios of >0.7 ms/s were detected. The upper and lower envelopes of the N–N interval variations were calculated as the 95th and 5th percentile points, respectively, within a sifting window with a width of 130 s. Then, the dips were defined as CVHR if the following criteria were met: (a) dip depth >40% of the envelope range; (b) inter‐dip interval (cycle length) 25–130 s; (c) average waveform morphological correlation coefficient >0.4 with the preceding and subsequent dips; and (d) the equivalence score of three cycle lengths between four consecutive dips >0.8. Fcv was calculated as the mean number of CVHRs per hour of time in bed.

### 2.5 Measurement of Acv

For all ECG data from the three databases, Acv was measured by the method of signal averaging according to our previous work (Hayano et al., 2017). Briefly, for all dips meeting the relaxed CVHR criteria (excluding criterion 4 from the criteria for Fcv), N–N interval segments around the nadir point of the dips were aligned at the nadir points and averaged. Acv was measured as the dip depth of the signal‐averaged interval curve, that is, the vertical distance from the nadir to the line connecting the local maxima on both sides of the dip. Because Acv was calculated for CVHR defined by the relaxed criteria, Acv could be computed even in the cases of zero Fcv.

### 2.6 Analysis of the correlates of Acv

The relationships between Acv, O_2_ desaturation, and sleep apnea severity were analyzed in the polysomnographic database. We generated scattergrams between Acv and O2 desaturation, between O_2_ desaturation and AHI, between AHI and Fcv, and between Fcv and Acv, and calculated Pearson's correlation coefficients for the associations.

### 2.7 Analysis of Acv–Fcv relationship map

The amplitude–frequency relationship of CVHR was analyzed by Acv–Fcv mapping using the ALLSTAR big data. Because Acv data showed different distributions depending on the value of Fcv, we employed a percentile regression of Acv by Fcv to model the characteristic distribution of Acv. The percentile values of Acv were determined within each bin of Fcv value. The bin width of Fcv was defined so that each bin contained 2,000 Acv data points, but if there were more than 2,000 of Acv data for a single Fcv value, then all such data points were included in that bin. Acv values were converted into the upper nearest integer percentile value between the 1st and 100th percentiles. To delineate the contours of Acv distribution, the Acv values with the same percentile were interpolated over the entire range of Fcv value.

### 2.8 Evaluation of predictive performance

The predictive performance was examined in the ENRICHD cohort data. The Acv values were converted into percentile Acv values by applying the percentile regression functions generated in the ALLSTAR database. The predictive performance was analyzed by logistic regression of mortality and evaluated by Somers' D and c‐statistic. All logistic models also included age as an independent predictor. Additionally, the improvement of predictive performance by the use of percentile Acv instead of unadjusted Acv was evaluated by net reclassification improvement (NRI) without category (continuous NRI; Pencina et al., 2008, 2011). The continuous NRI reflected the difference between the ratios of improvement and deterioration in predicted probability.

### 2.9 Statistical analysis

The SAS program package (SAS Institute) was used for these analyses. The standard error of c‐statistics was estimated by the bootstrap method with 1,000 random samplings. The significance of improvement in predictive performance was evaluated by the 95% confidence interval of continuous NRI that was also estimated by the bootstrap method with 1,000 random samplings. For all statistical analysis, *p* < .05 was considered significant.

## 3 RESULTS

### 3.1 Step 1: Correlates of Acv in polysomnogram database

**FIGURE 1:**
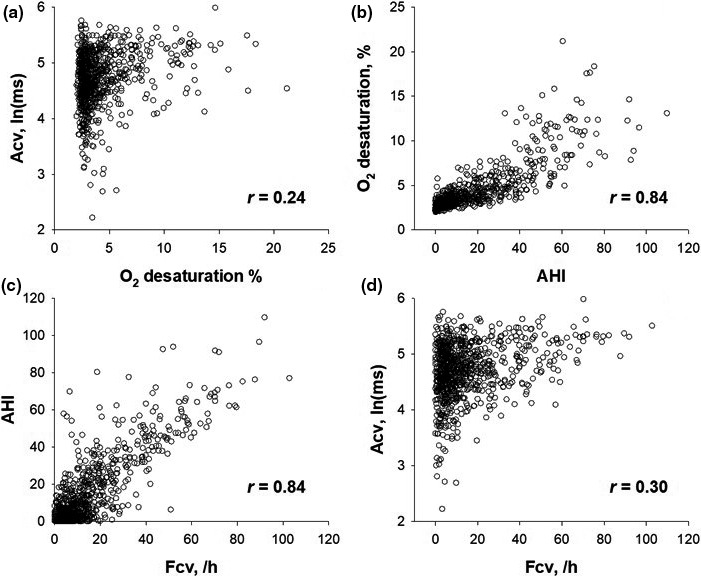
Relationships between the amplitude of cyclic variation of heart rate (CVHR), O_2_ desaturation, apnea‐hypopnea index, and the frequency of CVHR in patients undergoing diagnostic polysomnography for suspected sleep‐disordered breathing. Acv, amplitude of CVHR; Fcv, frequency of CVHR

Pearson's correlation coefficients showed significant associations between Acv, O_2_ desaturation, AHI, and Fcv. As shown in Figure 1a, the distribution of Acv depends on the level of O_2_ desaturation, with low Acv values likely to appear when O_2_ desaturation is mild. O_2_ desaturation correlated with sleep apnea severity assessed by AHI (Figure 1b), AHI correlated with Fcv (Figure 1c), and O_2_ desaturation correlated with Fcv (correlation coefficient, .77). Consequently, a decrease in Acv due to mild sleep apnea was also observed in the relationship between Acv and Fcv (Figure 1d).

### 3.2 Step 2: Acv–Fcv relationship mapping in the ALLSTAR database

**FIGURE 2:**
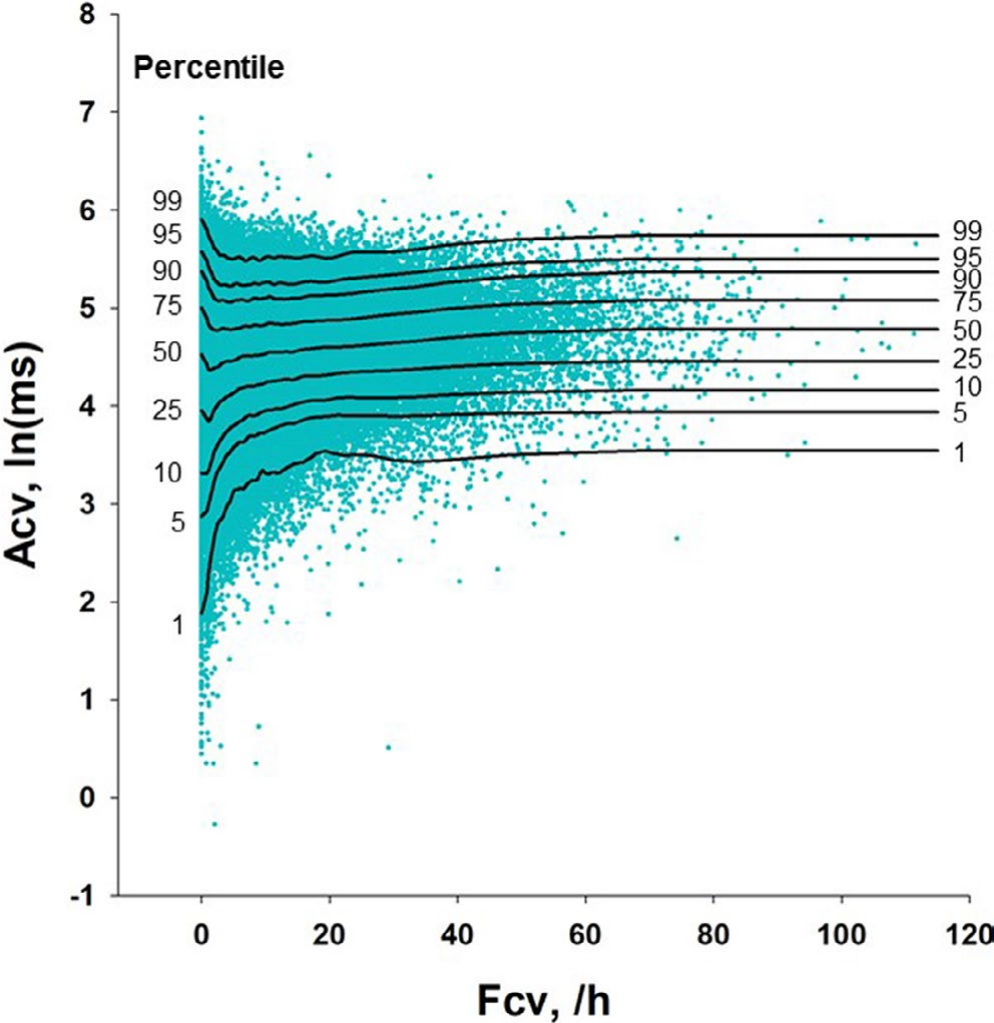
Distribution of Acv depending on the Fcv value and the percentile regression lines of Acv by Fcv in the ALLSTAR database. The percentile regression lines from 1st to 99th percentiles connect the respective Acv percentile points at the respective Fcv values

Among 405,911 ECG data in the ALLSTAR database, 194,490 ECG data that met both criteria were used. Participants were aged 65 ± 16 (mean ± *SD*) years and included 85,564 males and 108,926 females (Table 1). Figure 2 displays the distribution of Acv values depending on the Fcv value. The lower the Fcv value, the wider the distribution of Acv was from higher to lower values. The differences in Acv distribution with Fcv were clearly reflected by the contours of Acv percentile.

**TABLE 1:** Characteristics of participants in ALLSTAR database (*N* = 194,490)

Age, years	68 (57–77)
Female	108,926 (56.0%)
Heart rate, bpm	71.7 (65.2–78.2)
Fcv, /h	7.14 (3.68–12.6)
Acv, ln(ms)	4.52 (4.18–4.83)
Percentile Acv	50 (25–75)

Note

Data are median (IQR) or *n* (%).

Abbreviations: Acv, amplitude of cyclic variation of heart rate; Fcv, frequency of cyclic variation of heart rate.

### 3.3 Step 3: Acv–Fcv relationship and improvement of predictive performance in the ENRICHD cohort

**FIGURE 3:**
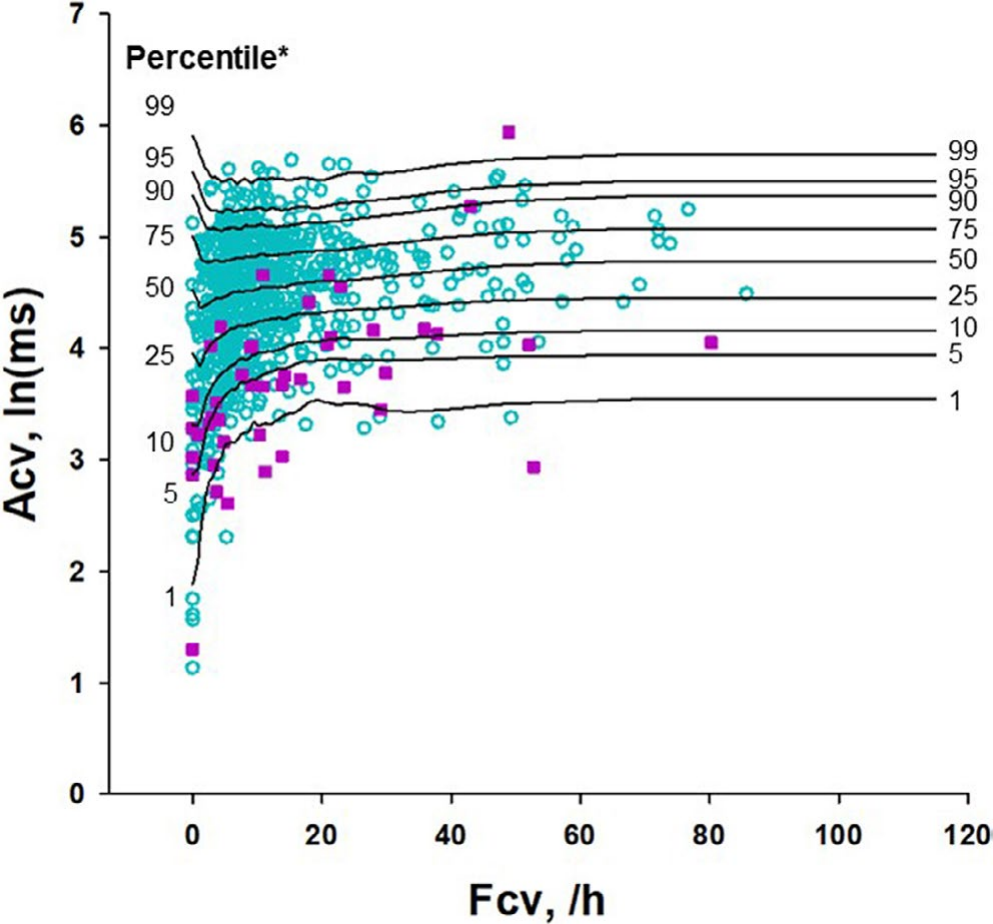
Distribution of Acv depending on the Fcv value in the ENRICHD cohort. Green circles represent survivors, and purple squares represent non‐survivors. The lines from 1st to 99th percentiles are the percentile regression lines that were generated in the ALLSTAR database (Figure 2)

The Acv values of both survivors and non‐survivors in the ENRICHD cohort showed similar distributions to those observed in the ALLSTAR database (Figure 3). The changes in Acv distribution by Fcv were consistent with the contours of Acv percentile obtained in the ALLSTAR database.

Table 2 shows the characteristics of survivors and non‐survivors in the ENRICHD cohort with the percentile Acv values. Both unadjusted Acv and percentile Acv were lower in the non‐survivors compared with the survivors, while Fcv did not differ significantly between two groups.

**TABLE 2:** Characteristics of patients in ENRICHD cohort (*N* = 717)

	Survivors *N* = 673	Non‐survivors *N* = 44	p*
Age, years	59 (50–67)	65 (56–73)	.001
Female	264 (39.2%)	20 (45.5%)	.4
Heart rate, bpm	67.6 (61.1–77.0)	72.2 (63.3–80.4)	.05
Fcv, /h	9.00 (4.79–15.5)	10.9 (3.63–23.1)	.4
Acv, ln(ms)	4.54 (4.08–4.86)	3.67 (3.23–4.08)	<.0001
Percentile Acv	48 (19–75)	7 (3–13)	<.0001

Note

Data are median (IQR) or *N* (%).

Significance of difference by Wilcoxon two‐sample test or chi‐square test.

Abbreviations are explained in the footnote to Table 1.

Table 3 presents the results of logistic regression analysis for all‐cause mortality risk. The percentile Acv showed the best predictive performance in terms of Somers’ D and c‐statistic, which was greater than the c‐statistic for Fcv (*p* = .0004) but was not significantly different from that of the unadjusted Acv. Compared with the unadjusted Acv, however, the percentile Acv showed a significant NRI of 0.90 (*SE*, 0.23; 95% CI, 0.51–1.42).

**TABLE 3:** Predictive power of Fcv, Acv, and percentile Acv for post‐AMI mortality in ENRICHD cohort (logistic regression analysis)

Predictor	Concordant, %	Discordant, %	Somers’ D	c‐statistic
Fcv	66.3	33.7	0.326	0.667 ± 0.041
Acv	81.5	18.5	0.630	0.817 ± 0.035
Percentile Acv	84.3	15.6	0.687	0.843 ± 0.030

Note

All models are adjusted for the effect of age.

## 4 DISCUSSION

Although it has been reported that a decrease in Acv is a powerful predictor of mortality risk after AMI, Acv could also decrease in mild sleep apnea, particularly when O_2_ desaturation is mild. In this study, we examined the hypothesis that the predictive power of decreased Acv could be improved by adjusting for the effects of the presence and severity of sleep apnea. Considering mortality risk stratification only by ECG data, we estimated the sleep apnea severity by Fcv. Analysis of ALLSTAR big data revealed that Acv showed different variances depending on the value of Fcv and that the lower the Fcv value, the wider the distribution of Acv from low to high values. We therefore used percentile regression functions to characterize the Acv–Fcv relationship and converted Acv into percentile values within each bin of the Fcv value. Based upon analysis of independent data from the ENRICHD post‐AMI cohort patients, the use of percentile Acv instead of unadjusted Acv resulted a significant continuous NRI of 0.90.

To our knowledge, this is the first study to demonstrate that the predictive power of blunted CVHR can be improved by considering the relation of Acv to sleep apnea severity. Previous studies reported that blunted CVHR is associated with an increased risk in cardiovascular diseases (Cao et al., 2019; Hayano et al., 2017), stroke (Noda et al., 1997), and end‐stage renal diseases (Hayano et al., 2017). However, none of these studies reported the impact of sleep apnea severity on the predictive power of blunted CVHR. This gap in our knowledge may be due partly to the characteristic distribution of Acv against sleep apnea severity that is not modeled appropriately by simple linear regressions. The lowest levels of Acv, however, were more likely to appear in participants with no or mild sleep apnea, as were the highest levels. To adjust for the effects of sleep apnea severity on Acv, we adopted percentile regression to model the Acv distribution in the Acv–Fcv relationship map.

One strength of the current study is the use of two independent datasets: one for the derivation of the prediction model (Acv percentile by Acv–Fcv mapping) and the other for evaluating the prediction performance of the model. We used the ALLSTAR big data as the derivation database, which can be expected to represent the general patient population undergoing Holter ECG monitoring in Japan (Hayano et al., 2018). Meanwhile, the ENRICHD data used for validation were obtained from post‐AMI patients from four clinical centers in diverse regions in the United States including the southeast, midwest, and northeast (Berkman et al., 2003). This study design may help prevent the predictive model from overfitting the study data and ensure the generalizability of the resulting model.

There are at least two mechanisms for the association between Acv and Fcv. First, polysomnographic analysis in patients with suspected sleep‐disordered breathing showed that sleep apnea episodes with mild O_2_ desaturation can cause a decrease in Acv (Figure 1a). Because O_2_ desaturation correlated with Fcv, Fcv and Acv also showed the association similar to that observed between O_2_ desaturation and Acv (Figure 1d). These observations suggest that the adjustment of Acv by Fcv may correct the overestimated mortality risk due to the decrease in Acv caused by mild O_2_ desaturation in patients with mild sleep‐disordered breathing. Second, a decrease in Acv can decrease Fcv because it can reduce the sensitivity to detect CVHR. If so, the increased appearance of low Acv values may be responsible for the lowering of Fcv. This mechanism, however, is unlikely to explain the reason why the adjustment of Acv by Fcv improves the predictive power of Acv.

One possible limitation of risk stratification by blunted CVHR is the requirement of the presence of sleep apnea episodes that are accompanied by CVHR. To address this issue, we used the relaxed criteria for CVHR only for the calculation of Acv. As a result, Acv was obtained in all participants both in the ALLSTAR database and in the ENRICHED cohort, including cases with zero Fcv. This method may have increased the risk of falsely recognizing nonspecific heart rate variability in the very low‐frequency band (0.0033–0.04 Hz) as CVHR. As shown in Figure 3, however, the Acv values even at zero Fcv were significantly lower in non‐survivors than in survivors (median [IQR], 3.28 [3.02–3.38] and 3.79 [3.21–4.39], *p* = .03).

## 5 CONCLUSIONS

Although a decrease in Acv has been reported to predict post‐AMI mortality risk, the analysis of ALLSTAR big data showed that Acv can also decrease with decreased Fcv. The adjustment of the effect of Fcv on Acv by the percentile regression model derived from the big data showed a significant improvement in the prediction of mortality in the ENRICHD independent post‐AMI cohort. This study indicates that the predictive power of Acv for post‐AMI mortality is improved by considering its relation to sleep apnea severity estimated by Fcv.

## CONFLICT OF INTERESTS

The authors have no conflict of interest to declare.

## AUTHOR CONTRIBUTIONS

J.H., E.Y., and E.W. conceptualized the study; J.H. contributed to methodology and software; E.W., E.Y., N.U., and M.K. validated the study; E.Y. involved in formal analysis; M.K. and N.U. investigated the study; R.M.C and J.A.B. (the ENRICHD study cohort), N.U., M.K., and J.H. (the ALLSTAR database), and E.W. (polysomnographic data) contributed to resources; N.U. curated the data; J.H. wrote—original draft preparation; J.A.B. wrote—review and editing; J.H. visualized the study; R.M.C. and J.A.B. supervised the study; J.H. and J.A.B. contributed to project administration; J.H., R.M.C., and J.A.B. involved in funding acquisition. All authors have read and agreed to the published version of the manuscript.

## Ethics

The study use of the all‐night polysomnogram database has been approved by the institutional review board of the Fujita Health University, Toyoake, Aichi, Japan (No. 09‐008), and by the Ethics Review Committee of the Nagoya City University Graduate School of Medical Sciences, Nagoya, Japan (No. 390). This study was a part of the Allostatic State Mapping by Ambulatory ECG Repository (ALLSTAR) project. The project has been approved by the Ethics Review Committee of Nagoya City University Graduate School of Medical Sciences (No. 709). The collection and analysis of Holter ECG recordings in the Enhancing Recovery in Coronary Heart Disease (ENRICHD) study were approved by the ethics committees of the corresponding clinical sites of the ENRICHD study (Washington University, St. Louis, Missouri; Duke University, Durham, North Carolina; Harvard University, Boston, Massachusetts; Yale University, New Haven, Connecticut). All participants provided written informed consent to participate in the study.

## Data Availability

The data that support the findings of this study are available on request from the corresponding author. The data are not publicly available due to privacy or ethical restrictions.
